# The effects of the general anesthetic sevoflurane on neurotransmission: an experimental and computational study

**DOI:** 10.1038/s41598-021-83714-y

**Published:** 2021-02-22

**Authors:** Jonathan Mapelli, Daniela Gandolfi, Enrico Giuliani, Stefano Casali, Luigi Congi, Alberto Barbieri, Egidio D’Angelo, Albertino Bigiani

**Affiliations:** 1 Department of Biomedical, Metabolic and Neural Sciences, University of Modena and Reggio Emilia, Sezione di Fisiologia e Neuroscienze, via G. Campi 287, 41125 Modena, Italy; 2 Center for Neuroscience and Neurotechnology, University of Modena and Reggio Emilia, 41125 Modena, Italy; 3 Department of Medical and Surgical Sciences for Children and Adults, University of Modena and Reggio Emilia, 41125 Modena, Italy; 4 Department of Brain and Behavioral Sciences, University of Pavia, 27100 Pavia, Italy; 5 Brain Connectivity Center, IRCCS Mondino Foundation, 27100 Pavia, Italy

**Keywords:** Cellular neuroscience, Molecular neuroscience

## Abstract

The brain functions can be reversibly modulated by the action of general anesthetics. Despite a wide number of pharmacological studies, an extensive analysis of the cellular determinants of anesthesia at the microcircuits level is still missing. Here, by combining patch-clamp recordings and mathematical modeling, we examined the impact of sevoflurane, a general anesthetic widely employed in the clinical practice, on neuronal communication. The cerebellar microcircuit was used as a benchmark to analyze the action mechanisms of sevoflurane while a biologically realistic mathematical model was employed to explore at fine grain the molecular targets of anesthetic analyzing its impact on neuronal activity. The sevoflurane altered neurotransmission by strongly increasing GABAergic inhibition while decreasing glutamatergic NMDA activity. These changes caused a notable reduction of spike discharge in cerebellar granule cells (GrCs) following repetitive activation by excitatory mossy fibers (mfs). Unexpectedly, sevoflurane altered GrCs intrinsic excitability promoting action potential generation. Computational modelling revealed that this effect was triggered by an acceleration of persistent sodium current kinetics and by an increase in voltage dependent potassium current conductance. The overall effect was a reduced variability of GrCs responses elicited by mfs supporting the idea that sevoflurane shapes neuronal communication without silencing neural circuits.

## Introduction

The selective interaction between general anesthetics and membrane proteins modulates synaptic transmission, membrane potential and signaling in neurons^1,2^. It is well established that the action of general anesthetics is characterized by a generalized neuronal hyperpolarization subtended by an increased inhibition or by a reduced synaptic excitation^1,2^. Among anesthetics, halogenated molecules are the most widely employed in medical practice. Nevertheless, their action mechanism is not yet fully understood, and their use is primarily governed by empirical rules. These molecules are allosteric modulators of synaptic receptors^3^. It has been shown in fact that they increase GABA-A and Glycine receptors activity^3^ whereas they typically downregulate the activity of cholinergic and NMDA-type glutamate receptors^4^. At the cellular level, halogenated anesthetics inhibit neuronal voltage-gated potassium^5^ and sodium channels^6,7^ and potentiate two-pore domain potassium channels^8^. As a side effect of their action, these anesthetics impair synaptic long-term potentiation hampering neuronal ability to store information^9^. In epileptic patients these drugs increase seizure activity^10^ and induce delirium and agitation during the recovery phases^11^. At the integrative level, the disruption of the information transfer among brain areas is supposed to be an essential step for the action of anesthetics^12^. The anesthesia could act by reducing the number of discriminable functional states in an integrated system as well as the complexity of the overall neural state^13^. Although these findings represent the state of the art in the knowledge of the effects of anesthetics at integrative level, a more detailed analysis of the changes in neuronal communication induced by anesthetics is still required.

The cerebellar cortical circuit is an ideal benchmark for the analysis of the effects of anesthetics on neurotransmission since GrCs show the unique characteristic among neurons of having a low number of dendrites (4.6 on average^14^), a very well detailed set of ionic channels and synaptic receptors, a compact electrotonic structure allowing stable electrophysiological recordings and the development of reliable computational models^15^.

While the cerebellum has long been considered a marginal target for general anesthetics^16^, it is now known that these cause (i) a reduction in PET and fMRI signals together with cortical and thalamic areas^17^ which participate with the cerebellum to peripheral sensory integration, (ii) a marked decrease of the frequency of spontaneous activity of the cerebellar cortex^18^, (iii) the appearance of coherent oscillations in the cerebellar cortex together with a decrease of the overall entropy of the system^19^. Recently, the cerebellum was shown to participate in cognitive^20^ and in sensory motor control^21^, furthermore, from consciousness to general anesthetics-induced unconsciousness, rich-clubs of nodes in functional brain networks are switched from the high-order cognitive function networks to sensory and cerebellum networks^22^. Compared with natural sleep, nodal efficiency of cerebellum (among others) significantly decreased during propofol-induced unconsciousness and functional connectivity between the cortex and subcortical centers (centralized in cerebellum) were significantly attenuated under sedation^23^. Therefore, the cerebellum is now turning out to be specifically involved in the process of consciousness regulation during anesthesia. The involvement of the cerebellum may reflect its strong connectivity with prefrontal and frontal areas^20^. The preferential reduction of low-frequency fluctuations in the anterior frontal regions and cerebellum is consistent with frontal to sensory-motor cortical disconnection and may contribute to the suppression of consciousness during general anesthesia^24^. Understanding the cellular mechanisms through which the cerebellum is modulated by general anesthetics could thus help understanding the mechanisms of induction and recovery from anesthesia.

The analysis of neurotransmission has been classically performed through experimental methods such as electrophysiology^25^, molecular biology^26^ and imaging^27^. More recently, the use of mathematical models to mimic the activity of neuronal circuits is increasingly becoming an efficient tool to predict brain dynamics^28^. Biologically realistic models can faithfully reproduce the electrical behavior of single neurons and synapses embedded in neural circuits performing computational tasks^29^. Despite the power of these methods, computational approaches are mostly employed in the analysis of large-scale networks^30^ whereas simulations are rarely employed to dissect microcircuits activity for pharmacological purposes.

Here, by using electrophysiological recordings and mathematical simulations, we investigated the cellular mechanisms underlying the changes induced by sevoflurane on neurotransmission between mossy fibers and granule cells at the input stage of the cerebellum.

## Results

**Figure 1:**
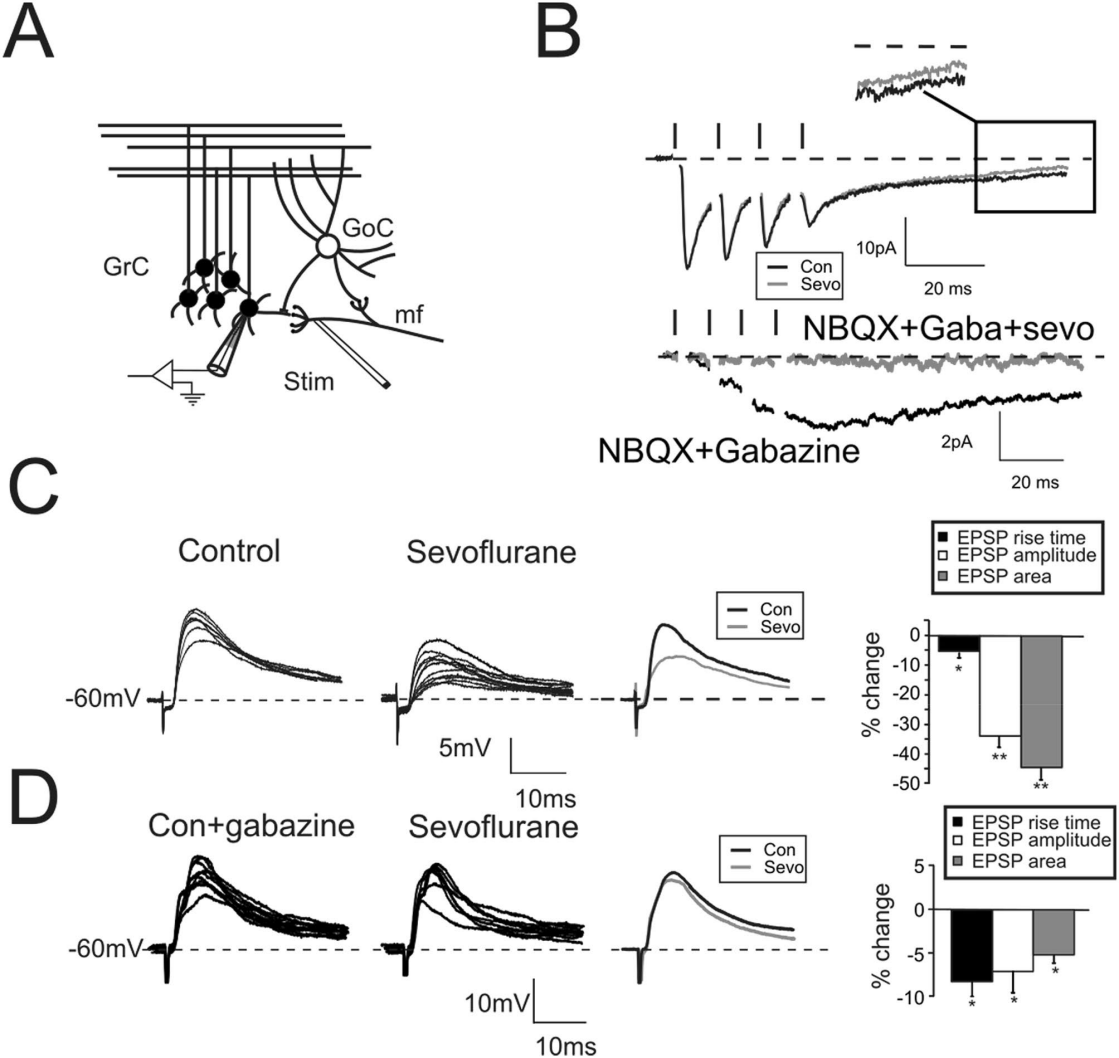
Modulation of excitatory neurotransmission by sevoflurane. (**A**) Scheme of the granular layer microcircuit. The stimulating electrode (stim) is positioned onto the mossy fiber bundle (mf) in order to activate excitatory synapses. GoC, Golgi cell (local interneuron); GrC, granule cell (output cell). (**B**) Top, EPSCs elicited in response to 4 pulses at 100 Hz and recorded from a GrC voltage clamped at − 70 mV (n = 9). Note the effects on the residual current induced by sevoflurane (gray trace, box). Bottom. In a different cell EPSCs were evoked from a GrCs voltage clamped at − 40 mV and in the presence of gabazine and NBQX (n = 8). Sevoflurane completely abolished the NMDA current. (**C**) EPSPs elicited by sub-threshold stimuli in GrCs at – 60 mV (10 superimposed traces) in control conditions (left) and in the presence of sevoflurane (middle). The average traces (right) show the marked decrease of EPSP amplitude and area induced by sevoflurane (gray trace). Histogram summarizes the effects induced by sevoflurane on EPSP rise time, amplitude, and area (n = 7). In this and in the following figures: *p < 0.05; **p < 0.01. (**D**) EPSPs were recorded from GrCs at – 60 mV (10 superimposed traces) and in the presence of gabazine (top traces) to unmask the NMDA component (n = 4) in control (left) and in the presence of sevoflurane (middle). The average traces (right) show that sevoflurane decreases both peak amplitude and EPSP area. Histogram summarizes the effects induced by sevoflurane on EPSP rise time, amplitude, and area.

In the cerebellar cortex, information from mossy fibers (mf) activate granule cells (GrCs) and Golgi cells (GoCs) through glutamatergic synapses. GoCs, which are also excited by feedback loops from GrCs, inhibit the same GrCs through GABAergic synapses (Fig. 1A). A similar circuit architecture with a functional organization composed by reciprocal excitatory and inhibitory connections can be found in various central and peripheral neural circuits^31^. We have employed the cerebellar micro-circuitry as an experimental model of the information processing in the CNS to investigate the impact of sevoflurane on neurotransmission.

### Sevoflurane inhibits excitatory neurotransmission on granule cells

Excitatory Post-Synaptic Currents (EPSCs) were recorded from GrCs voltage clamped at -− 70 mV in response to mf bundle stimulation (Fig. 1A). The activation of inhibitory loops through Golgi cells did not produce significant inhibitory currents because the reversal potential of chloride was about − 60 mV^32,33^. In response to a 4-pulse, 100-Hz burst (Fig. 1B), EPSCs showed the typical short-term depression pattern^34^, composed of a rapid AMPA and a slow NMDA component. The application of sevoflurane did not significantly affect EPSCs peak amplitudes (1st peak change + 3.5 ± 1.8%, *p* > 0.35; n = 5, Fig. 1B). This result is in accordance with our recent findings on desflurane, a chemical compound of the same family of sevoflurane, showing that glutamate AMPA receptors are not targeted by the anesthetic^33^. When membrane potential is lower than − 40 mV, the voltage-dependent magnesium block is expected to dampen NMDA currents. However, a residual component of this glutamatergic current can be effectively detected at more hyperpolarized values^34^. This late component is unmasked by measuring the amount of excitatory currents 50 ms after the end of the stimulation pattern (Fig. 1B top, box) and sevoflurane indeed reduced the residual NMDA component of NMDA current (− 27.8 ± 2.3%, *p* < 10^–5^; n = 9, Fig. 1B). In order to further isolate NMDA currents, GrCs were voltage clamped at − 40 mV and the Mg^2+^ was removed. In the presence of both AMPA (NBQX) and GABA-A receptor (Gabazine) blockers (Fig. 1B, bottom traces) currents showed a marked temporal summation peaking in about 20 ms after the last stimulus (− 2.6 ± 0.2 pA n = 8; at the peak of the current). This effect was completely abolished by the application of sevoflurane (− 95.5 ± 2.1% n = 8; *p* < 10^–5^) supporting the evidence that NMDA channels are indeed targeted by the anesthetic^4^. In addition, sevoflurane rapidly and transiently reduced Excitatory Post-Synaptic Potentials (EPSPs) both in peak amplitude (− 33.9.1 ± 3.5%, *p* < 0.001, n = 7, Fig. 1C) and in total depolarization (EPSP area − 45.4 ± 7.2%, *p* < 0.01 n = 7, Fig. 1C). The NMDA current is known to favor the temporal summation of concomitant inputs leading to a sustained membrane depolarization. We therefore evaluated the impact of NMDA blocking induced by sevoflurane onto the generation of EPSPs (Fig. 1C, lower traces) in the presence of gabazine. Unexpectedly, peak amplitude (− 6.9 ± 2.3%, *p* < 0.05, n = 4, Fig. 1D) and EPSP area (− 5.1 ± 0.9%, *p* < 0.05, n = 4, Fig. 1D) were only slightly reduced indicating that, although the excitatory neurotransmission was affected by the block of the NMDA current, the anesthetic mostly impacted the inhibitory component of neurotransmission. Interestingly, in both cases, we observed an acceleration of the rise time of EPSP indicating changes in the overall kinetics of the synaptic machinery (Fig. 1D, histograms).

**Figure 2:**
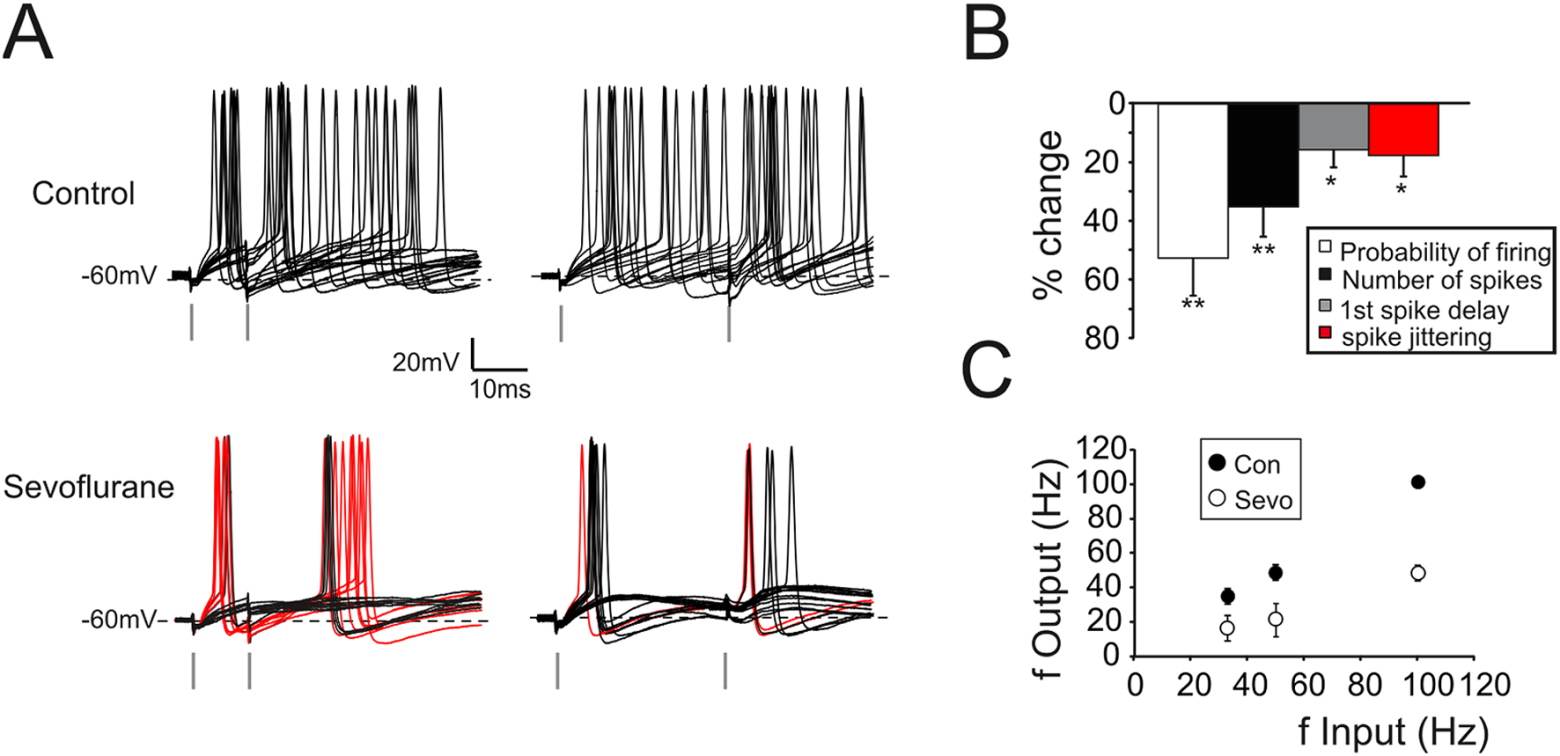
Modulation of GrCs firing activity by sevoflurane. (**A**) Top. Spikes from GrC elicited in response to a pair of stimuli at 100 Hz (left) and 30 Hz (right) (15 superimposed traces). Bottom. Sevoflurane reduces the total number of spikes and the probability of firing in response to the first stimulus. Note that the first spike is anticipated and elicited with a less variable delay. Red traces show responses in which stimuli elicited two spikes. (**B**) Histogram summarizes the effects induced by sevoflurane on spike related parameters (n = 7). (**C**) The plot shows the relationship between input frequency and output frequency in control (Con) and during sevoflurane perfusion (Sevo) (n = 7).

One of the mathematical relationships that better describes neurotransmission is that between input and output variables (I/O). In response to pairs of action potentials delivered by mf at variable frequencies^32^, GrCs responded with two or more spikes (Fig. 2A,B) with a quasi-linear relationship (Fig. 2C). The presence of sevoflurane profoundly altered the GrCs I/O by reducing the probability of eliciting spikes (− 52.8 ± 14.3%, *p* < 0.01; n = 7; Fig. 2C), as well as the total number of emitted spikes (− 35.2 ± 9.1%, *p* < 0.01; n = 7; Fig. 2C). Furthermore, GrCs mostly responded to low frequency inputs with EPSPs or at most single spikes (44 ± 2.9% singlet; 54.3 ± 3.4% EPSPs over total responses at 33 Hz; Fig. 2B bottom, black traces n = 7). Only in few cases, doublets of action potentials were generated (2.9 ± 1.8% doublets over total responses at 33 Hz; Fig. 2B bottom, red traces n = 7) while the average firing frequency was markedly reduced (− 58.6 ± 12.2% at 100 Hz, *p* < 0.01; n = 7; Fig. 2A–C). The overall result was a downward shift of the frequency dependence curve in accordance with the reduction of NMDA currents and with the increased GABAergic inhibition. Surprisingly, the first spike delay and its variability were reduced (delay: − 15.7 ± 5.9%, *p* < 0.05; n = 7; Fig. 2C; variability: − 17.7 ± 8.1%, *p* < 0.05; n = 7; Fig. 2C).

### Sevoflurane potentiates GABAergic neurotransmission on granule cells

The large majority of anesthetics, including halogenated ones affects neurotransmission by potentiating GABAergic currents^3,33^. We therefore evaluated the effect of sevoflurane on the GABAergic synapse whose activity was monitored by voltage clamping GrCs at 0 mV. The GABAergic currents were identified as positive current deflections^32,33^ elicited by the direct stimulation of GoC axonal plexus (Fig. SM-1A). Inhibitory currents were pharmacologically isolated by adding 20 μM NBQX and 50 μM D-APV to the extracellular solution to block excitatory neurotransmission and preventing the activation of poly-synaptic pathways (see Fig. SM-1A). Spontaneous Inhibitory Post-Synaptic Currents (sIPSCs), which were present in almost all recordings (13/14 cells) occurred at an average frequency of 3.1 ± 0.6 Hz (Fig. SM-1B, n = 13). Stimulation of GoC axons with two pulses at 50 Hz (see Materials and Methods) elicited pairs of evoked Inhibitory Post-Synaptic Currents (eIPSCs; Fig. SM-1C) that were abolished together with sIPSCs following the perfusion of 10 μM gabazine (Fig. SM-1C inset; n = 5). These results also confirmed the absence of slow GABA-B receptor-mediated responses in granule cell inhibitory currents. The time courses of sIPSCs and eIPSCs shared similar kinetics both for the current rise (sIPSC rise_10–90_ 1.39 ± 0.26 ms, n = 13; eIPSCs rise_10–90_ 1.45 ± 0.46 ms, n = 13) and for the decay component (sIPSCs τ = 17.8 ± 4.7 ms; n = 13 Fig. SM-1D black trace; eIPSCs τ = 18.3 ± 4.1 ms; n = 13, Fig. SM-1D gray trace), confirming that the stimulation protocol elicited currents similar to the ones spontaneously evoked by Golgi cells. A sustained slow decay component could be also observed in eIPSCs, consistently with an indirect receptors’ activation through GABA spillover into the cerebellar glomerulus^35^ and this slow current was further unmasked by repetitive stimulation (Fig. SM-1D, red trace).

**Figure 3:**
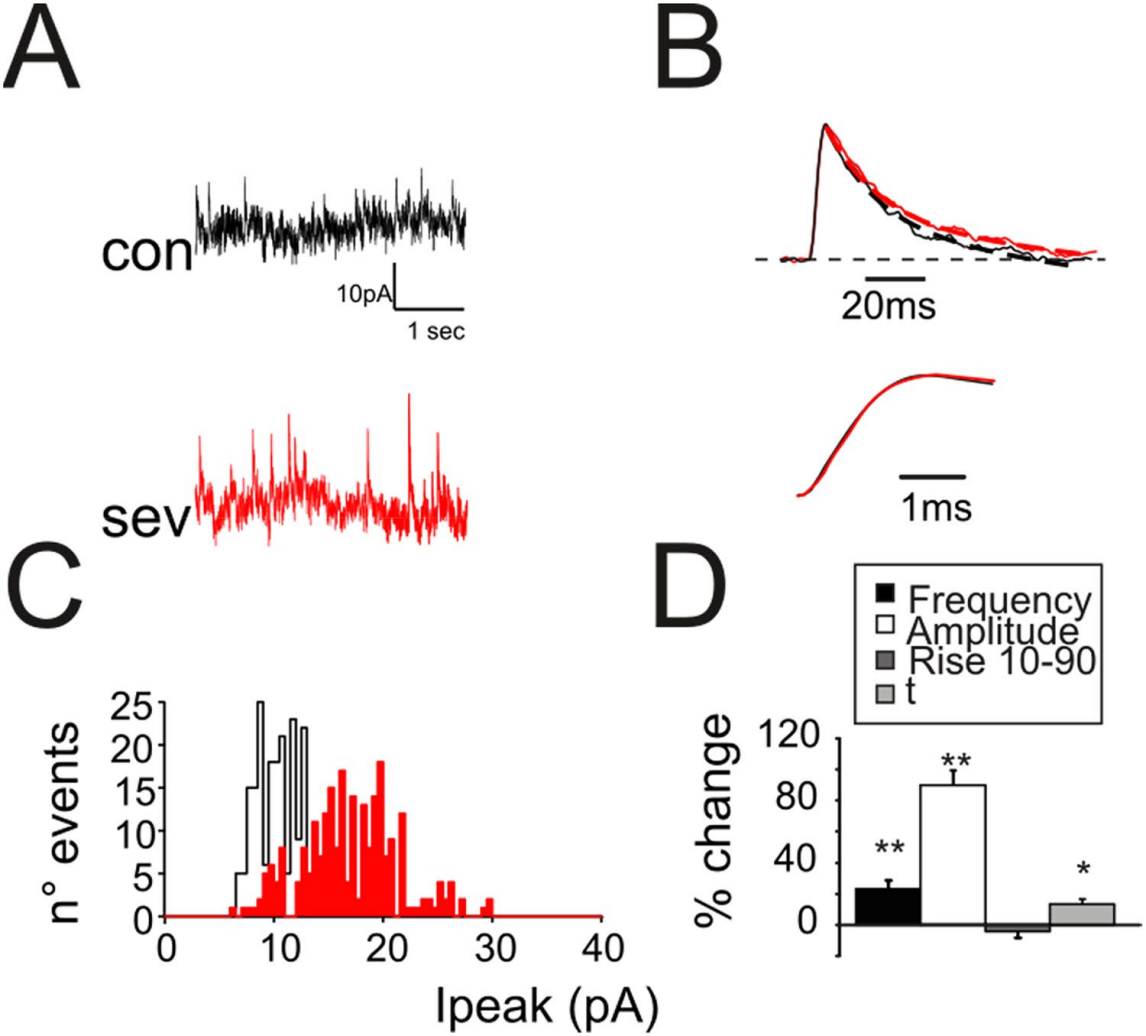
Modulation by sevoflurane of spontaneous inhibitory synaptic currents. (**A**) sIPSCs recorded from a granule cell before (black) and after (red) the application of sevoflurane. Note the increased frequency and peak amplitude. (**B**) Normalized sIPSCs recorded from a granule cell before (black) and during (red) sevoflurane application. The mono-exponential fitting (dashed lines) of the current relaxation reveals small changes in decay. Bottom: rise time of the sIPCSs shown in the upper panel. (**C**) Distribution of sIPSCs peak amplitudes detected during a recording of 3 min in control condition (black histogram) and in the presence of sevoflurane (red). (**D**) Histogram shows the effects induced by sevoflurane on sIPCS biophysical properties (n = 13).

In contrast with the effect induced by desflurane^33^, sevoflurane altered spontaneous IPSCs by increasing frequency (+ 25.5 ± 4.9%, n = 7; *p* < 0.01; Fig. 3A,D) and peak amplitude (+ 87.5 ± 9.6%, n = 7; *p* < 0.01, Fig. 3A,D). Furthermore, the presence of anesthetic did not significantly change sIPSC rise time (rise_10–90_; − 3.9 ± 2.7%, n = 7; *p* > 0.35, Fig. 3B bottom traces, Fig. 3D) while slowed down the current decay (τ = 15.4 ± 4.9% n = 7; *p* < 0.05 Fig. 3B top traces, Fig. 3D) indicating that the anesthetic modified the GABAergic synaptic complex.

**Figure 4:**
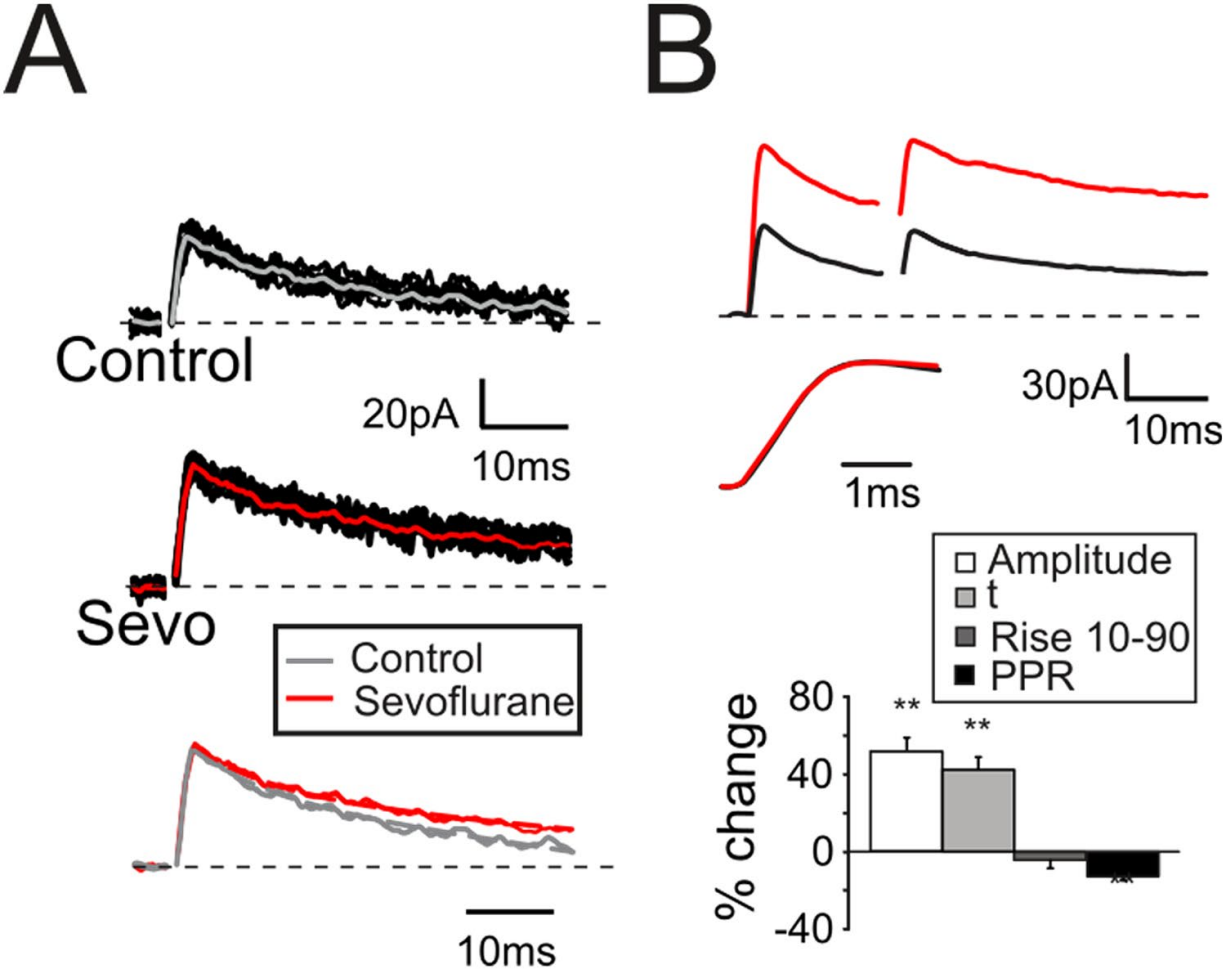
Modulation by sevoflurane of evoked inhibitory synaptic currents. (**A**) eIPSCs elicited by a single stimulus and recorded from a GrC in control conditions (top 20 superimposed traces) and during the application of sevoflurane (middle 20 superimposed traces). Note the increased peak amplitude and the slower decay. Bottom: normalized averaged eIPSCS. (**B**) eIPSCs elicited by a pair of stimuli at 50 Hz and recorded from a GrC before (black) and during (red) the application of sevoflurane. Middle traces: rise time of the eIPSCs shown in the upper panel. Histogram summarizes the effects induced by sevoflurane on the eIPSCS biophysical properties (n = 11).

The analysis of eIPSCs confirmed that sevoflurane, according to the observations for sIPSCs, increased both peak amplitude (+ 47.6 ± 7.1%, n = 11; *p* < 10^–4^, Fig. 4A,B top traces and histogram) and decay time course (τ = + 43.8 ± 6.5%, n = 11; *p* < 10^–3^, Fig. 4A bottom traces and Fig. 4B histogram) while the rise time was unaffected (rise_10–90_; − 3.9 ± 2.5%, n = 11; *p* > 0.4, Fig. 4B middle traces). Finally, together with the increase in sIPSC frequency and peak amplitude, the reduction of the eIPSC PPR (− 12.7 ± 2.3%, n = 11; *p* < 10^–3^, Fig. 4B top traces and histogram) suggested a modification in the presynaptic release machinery. As a whole, these results indicated that sevoflurane induced an increase in vesicle release probability as well as a change in post-synaptic receptor activity by increasing the total transferred charge evaluated as IPSC area (+ 109.1 ± 17.9%, n = 11; *p* < 10^–3^, data not shown).

### Sevoflurane increases intrinsic excitability of granule cells

The neuronal firing is primarily dependent on the ratio between excitatory and inhibitory input but is also tightly bound to ionic mechanisms bringing membrane potential to spike threshold. We have investigated the role of sevoflurane on GrC intrinsic excitability by collecting GrCs voltage responses to current injections in current clamp configuration, in the presence of 50 mM D-APV and 20 mM NBQX to block the contribution of glutamatergic afferences and 20 mM gabazine to discard the contribution of GABergic inhibition. The zero-current potential, which can give an estimate of resting membrane potential in patch-clamp experiments^36^, was monitored throughout the recordings: no significant variations could be observed during sevoflurane perfusion (Table SM-1).

**Figure 5:**
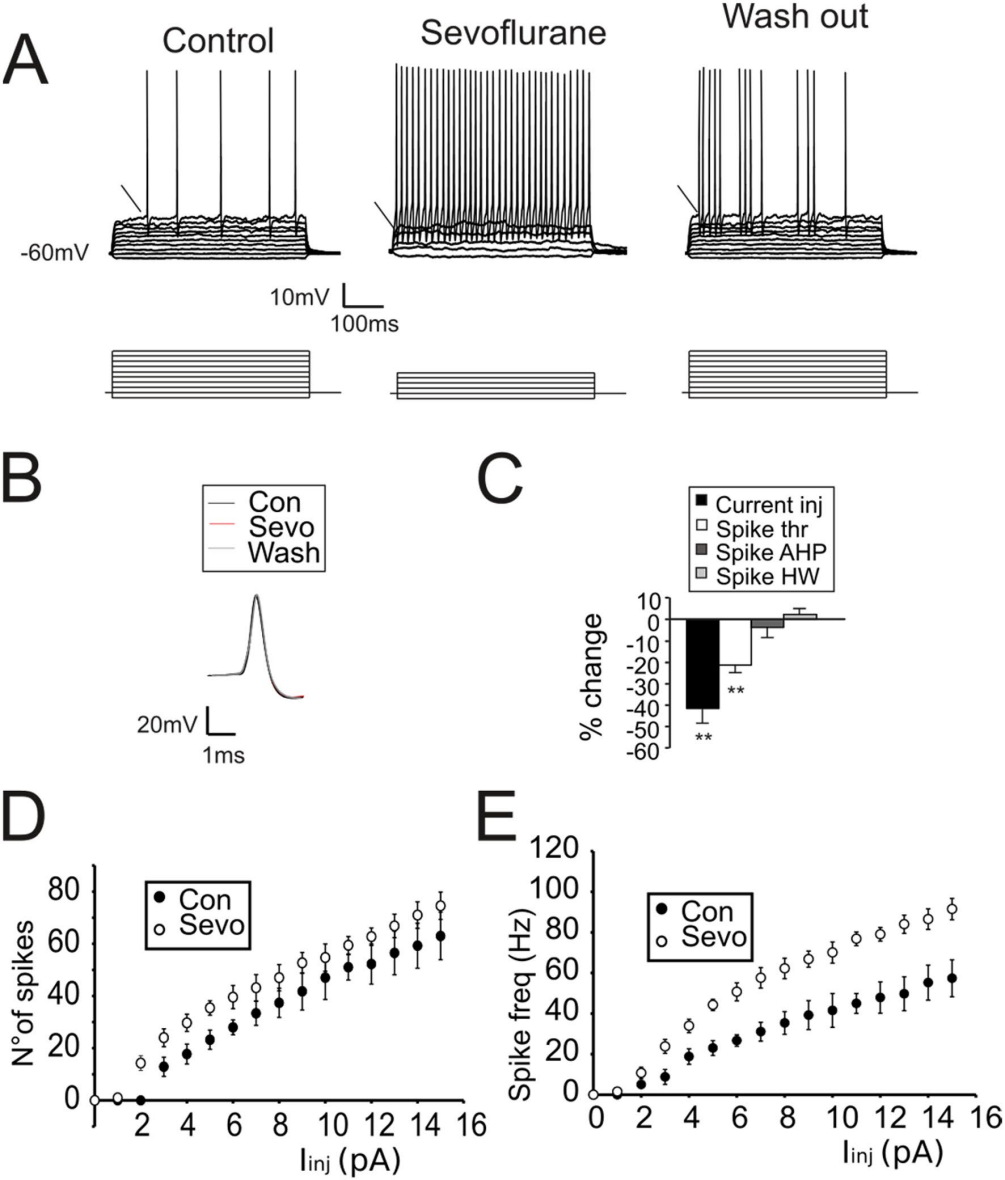
Sevoflurane increases GrCs intrinsic excitability. (**A**) GrC voltage responses to current injections (bottom traces 1 pA/step) in control conditions, during sevoflurane perfusion and following wash-out of the anesthetic. Note, during sevoflurane, less injected current is needed to generate action potentials, the number of elicited spikes is increased, the firing threshold is lowered (arrow) and firing discharge becomes regular. (**B**) Comparison of action potential waveform obtained in control (black), in the presence sevoflurane (red trace) and following wash-out (gray trace). Note that sevoflurane did not affect the spike shape. (**C**) Histogram shows the changes induced by sevoflurane on the current needed to bring GrCs to the firing zone (current inj), spike threshold (spike thr), spike after hyperpolarization (spike AHP) and spike half-width (spike HW) (n = 7). (**D**) Relationship between the injected current and the total number of emitted spikes (n = 7 cells). E Relationship between the injected current and the average firing frequency of the emitted spikes (n = 7 cells).

In response to depolarizing current injection, GrCs generated repetitive spike discharges (Fig. 5A, Control). During sevoflurane perfusion, the current needed to generate action potentials was significantly reduced (from 5.9 ± 0.8 pA in control, to 4.1 ± 0.8 pA with sevoflurane, *p* < 0.01 n = 7; Fig. 5C) by virtue of a spike threshold decrease (− 46.5 ± 2.9 mV in control and − 56.3 ± 3.1 mV in sevoflurane, n = 7 *p* < 0.01; Fig. 5C). Moreover, an increased number of emitted spikes (+ 68.3 ± 15.9%, *p* < 0.01, n = 7; not shown in the histogram) together with enhanced average (Fig. 5A; + 25.2 ± 7.1%, *p* < 0.05, n = 7; not shown in the histogram) and instantaneous firing frequency (Fig. 5A; + 46.7 ± 6.8%, *p* < 0.01, n = 7; not shown in the histogram) supported the idea that sevoflurane could alter the GrC intrinsic excitability, despite leaving unaffected spike waveform (Fig. 5B). These findings are summarized by the plot representing the relationship between the injected current and the number of emitted spikes and the average firing frequency (Fig. 5D,E).

**Figure 6:**
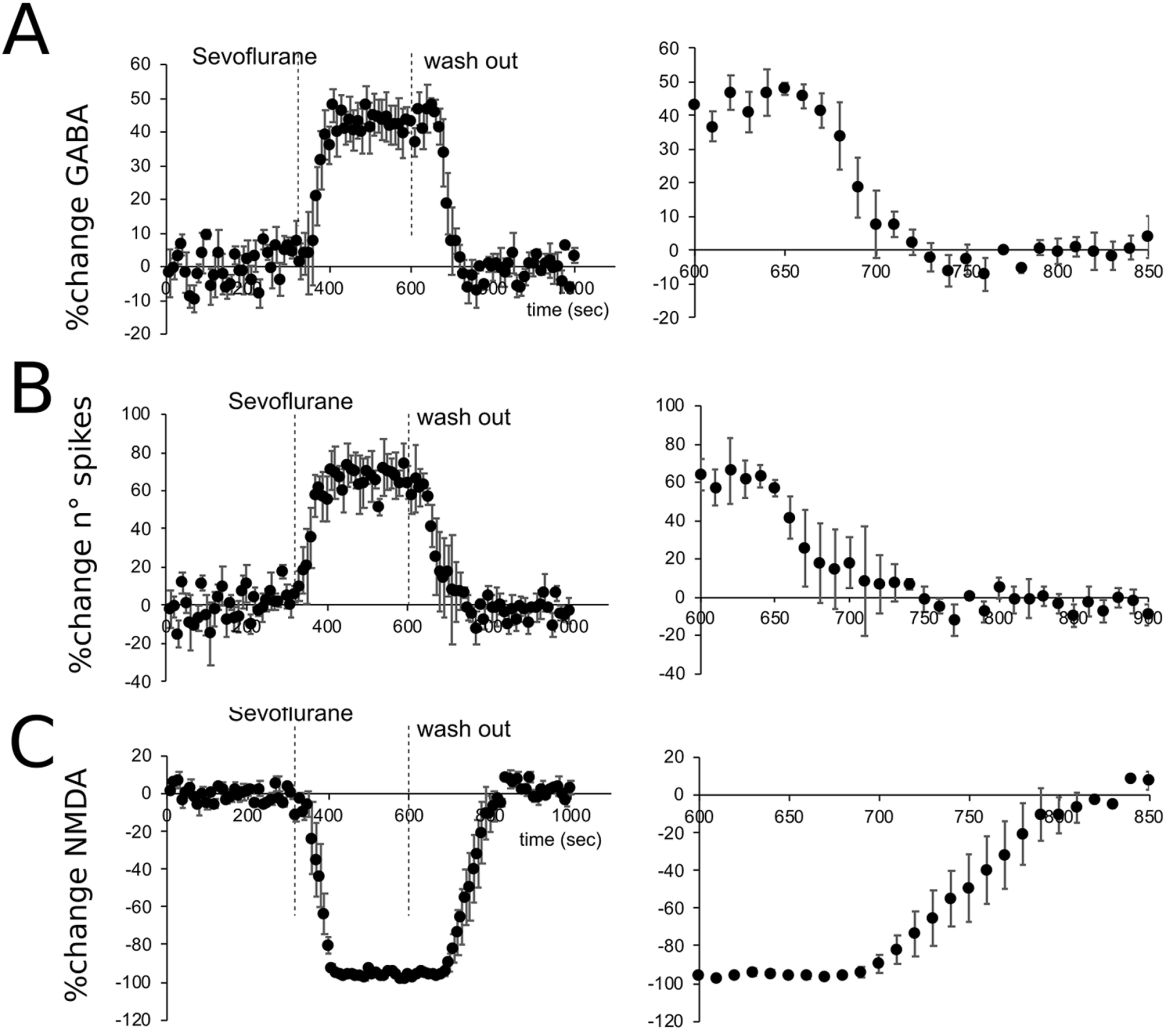
Time courses of the effect of sevoflurane. (**A**) Left; Time courses of the effect of sevoflurane (1st vertical dashed line 300 s) and subsequent wash out (2nd dashed line 600 s) on eIPSCs peak amplitude changes. Right. Inset of the panel shown on the left. Note that the return to the initial level starts 50 s after the beginning of washout and the steady state is obtained in about 100 s. (**B**) Left Time courses of the effect of sevoflurane on the number of spikes evoked in response to a 500 ms 5 pA depolarizing step current. Right. Inset of the panel shown on the left. Note that the return to the initial level starts about 40 s after the beginning of washout and the steady state is obtained in about 100 s. (**C**) Left Time courses of the effect of sevoflurane on the NMDA peak current changes. Right. Inset of the panel shown on the left. Note that the return to the initial level starts about 100 s after the beginning of washout and the steady state is obtained in about 200 s.

An important aspect of the action of anesthetics is the kinetics which heavily impacts the recovery processes. We have therefore investigated the time courses of the action of sevoflurane by eliciting GABAergic or NMDA currents every 10 s and monitoring currents properties during sevoflurane perfusion and during the subsequent wash-out (Fig. 6). On average, GABAergic currents started to increase around 30 s after the beginning of the perfusion (Fig. 6A, left; n = 4) and reached the steady state level in about 100 s (Fig. 6A, left; n = 4). In response to the anesthetic wash out, GABAergic currents started to decrease after about 50 s (Fig. 6A, right; n = 4) while the initial conditions were restored after 150 s. Similarly, the number of spikes generated by GrCs in response to current injection was monitored throughout perfusion and washout procedures (Fig. 6B). The plateau of variation, which started in less than 50 s, was reached in about 100 s (Fig. 6B left, n = 4), whereas the recovery phase started 50 s after the beginning of wash out and terminated after 100 s (Fig. 6B right, n = 4).

Conversely, NMDA peak currents showed slower kinetics both for the perfusion (50 s for the initial decrease and more than 130 s to steady state level, Fig. 6C left; n = 4) and wash out phase (100 s to start the recovery ad 250 s to restore the initial conditions, Fig. 6C right, n = 4).

### Modeling the effect of sevoflurane on neurotransmission

In order to understand the mechanisms underlying the changes in the intrinsic excitability caused by sevoflurane, we have employed a mathematical model of the GrC derived from previous versions^37,38^ that incorporates a detailed representation of all the expressed ionic conductance (see Materials and Methods). According to experimental observations, the simulations showed that GrCs responded to injected depolarizing current with repetitive spike discharges arising at − 47 mV.

The altered excitability observed in the presence of sevoflurane was mimicked by analyzing the impact of the ionic channels that are mainly involved in the regulation of spike threshold (Na_v_, K_v_ and leakage). Preliminarily, the contribution of all other channels which are known to be expressed in cerebellar GrCs and are incorporated in the model^37,38^ have been explored and none of the tested conditions could reproduce the experimental observations (data not shown).

**Figure 7:**
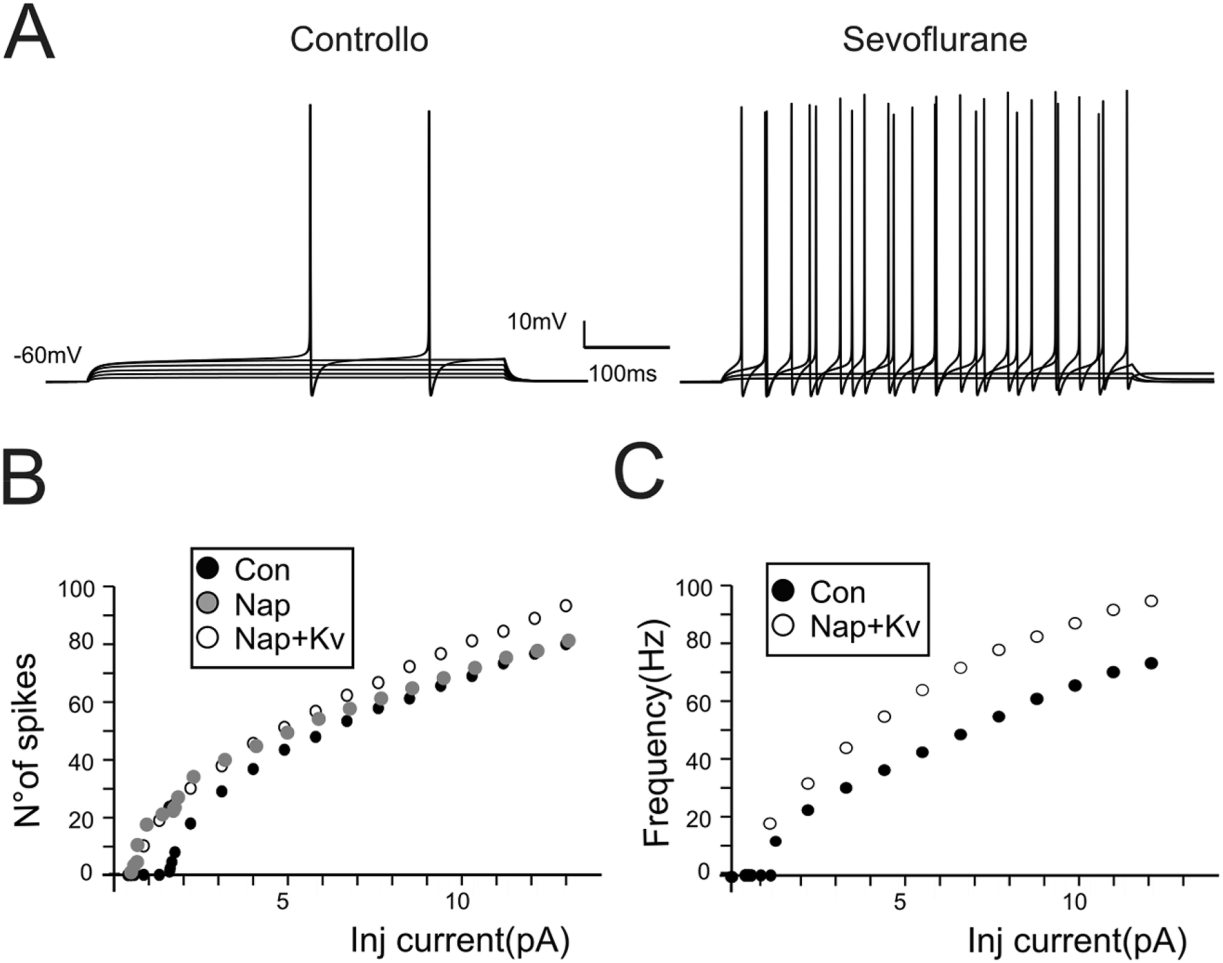
Simulation of GrCs intrinsic excitability. (**A**) Simulated GrC voltage responses to current injections (1 pA/step) in control conditions (left panel) and mimicking the presence of sevoflurane (right panel) by increasing the persistent sodium conductance (Nap) and voltage dependent potassium conductance (Kv). Note that, as observed experimentally, less injected current is needed to generate action potentials with sevoflurane. Also note the increased number of elicited spikes and the reduced firing threshold in the presence of sevoflurane. (**B**) The plot shows the number of spikes generated in response to current injection in control, when Nap is increased, and when both Nap and Kv are increased. Note that simulations with increased Nap tend to saturate more rapidly than those obtained when both Nap and Kv are increased. (**C**) The plot shows the relationship between the current injected and the average firing frequency in control condition and when both Nap and Kv are increased.

The altered excitability observed in the presence of sevoflurane was therefore modelled by changing the different components of the voltage dependent sodium channels (see Materials and Methods), which are known to be involved in the modulation of spike threshold. The increase of both activation and deactivation kinetics of the persistent component of Na^+^ current—Nap—(A_on_ from 0.75 to 1.5 ms and A_off_ from 0.005 to 0.05 ms, Fig. 7A) allowed to lower the GrC firing threshold (from − 47 to − 54.8 mV, Fig. 7A). Furthermore, by measuring the voltage response to current injection (Fig. 7C), the model reliably reproduced the responses obtained experimentally (cfr Fig. 5E). However, the analysis of I/O curve, in terms of number of spikes (Fig. 7B) and average firing frequency (Fig. 7C), revealed that changes of Na_p_ kinetics could not explain the overall GrC behavior observed experimentally. In particular, while GrCs tended to show a linear increase in the difference between the number of spikes in control and in the presence of sevoflurane at increasing current injections (Fig. 7C), the sole modifies in Na_p_ kinetics induced a saturation of the difference between curves at large current injection (Fig. 7C, gray circles). Conversely, by increasing the overall conductance of voltage dependent Na current (0.03 S/cm^2^ to 0.04 S/cm^2^ in the hillock and 0.02 S/cm^2^ to 0.03 S/cm^2^ in the axon) the spike discharge behavior approached the one observed experimentally (Fig. 7A,C). Finally, the changes induced in the spike overshoot and after hyperpolarization were compensated by increasing the overall potassium conductance (from 0.003 to 0.005 S/cm^2^ both in the hillock and in the axonal compartments).

**Figure 8:**
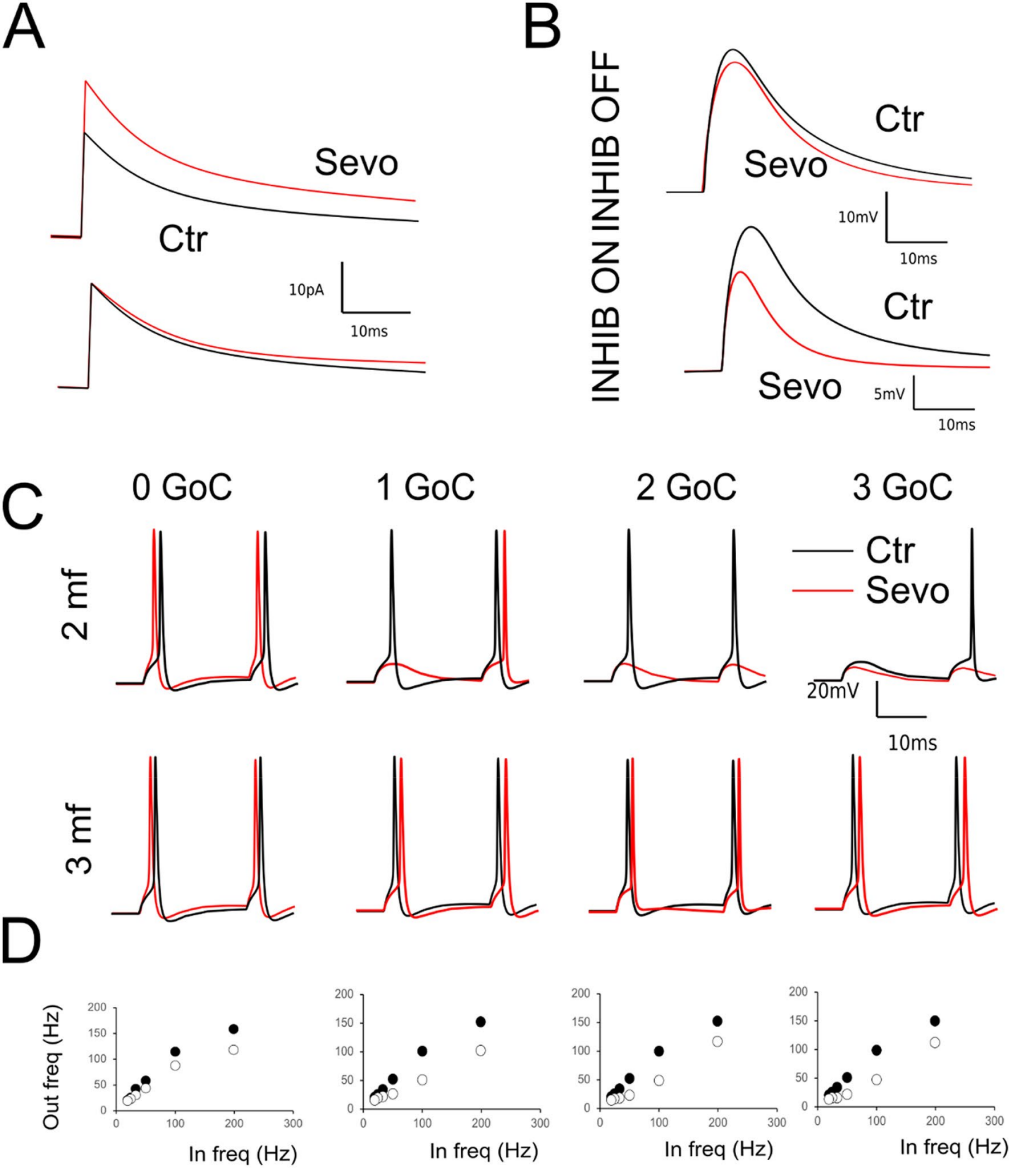
Simulation of synaptic activity. (**A**) (Top) IPSCs generated by a single stimulus in control (black) and mimicking the presence of sevoflurane (red). (Bottom) Normalized IPSCs show an increase in the current tail resulting from changing postsynaptic parameters. (**B**) (Top) EPSPs generated by activating a single mossy fiber in control condition (black) and mimicking the presence of sevoflurane (red). Note the reduction of the late EPSP phase caused by NMDA receptor block. (Bottom) EPSPs generated by activating three mfs and a single GoC in control condition and mimicking the presence of sevoflurane. (**C**) The effect of sevoflurane on GrCs is shown in terms of spike discharging at different E/I combinations in control (black) and in the presence of sevoflurane (red). Note that in the presence of active GoCs, sevoflurane decreases the number of spikes and posticipates spike generation. Conversely spikes are anticipated in case of null inhibition. (**D**) I/O curves referred to responses obtained with 3 mf and a variable number of GoCs (from 0 to 3) were obtained changing the stimulation frequency from 10 to 200 Hz simulating control conditions (black circles) and mimicking the presence of sevoflurane (white circles).

The effects of sevoflurane on cerebellar neurotransmission were further explored by incorporating changes in conductance into the GrC model and investigating the synaptic parameters space. The GABAergic currents were reproduced by simulating the intracellular recordings of chloride currents in GrC at a holding potential of − 60 mV activated by a single GoC (Fig. 8A). The changes observed experimentally (see Figs. 3, 4, 5) were reliably reproduced by modifying the parameters accounting for both pre- and post-synaptic activity (see Table SM-2). Given these changes on the inhibitory synapses, we have simulated the effects of sevoflurane on neurotransmission (Fig. 8A,B) by removing NMDA conductance, potentiating GABAergic currents and altering the GrC intrinsic excitability in a simplified version of the cerebellar microcircuit. According to anatomical findings^39^, a reduced version of the granular layer circuitry was assembled by connecting the GrC with a variable number of excitatory mfs connections and Golgi Cells (from 1 to 4 and from 0 to 7 respectively^40^). Similarly, to experimental observations, IPSCs evoked by single stimuli were increased in peak amplitude (+ 46.3%) and in the total transferred charge (+ 99.6%) by GABAergic currents (Fig. 8A. Furthermore, we simulated the generation of EPSPs in the presence and without the activity of inhibitory currents. Also, simulations reliably reproduced the behavior observed experimentally. The EPSPs were in fact markedly decreased in peak amplitude (− 32.4%) and total depolarization (− 57.1%) when GABAergic synapses were active while the effect of sevoflurane was barely measurable when inhibition was disactivated (− 8.9% peak amplitude, − 10.2% total depolarization, Fig. 8B).

Unlike experimental conditions, where the number of excitatory and inhibitory afferences can only be tentatively estimated, we have explored different combinations of mfs and GoCs to evaluate the parameters affecting neurotransmission, such as first spike delay, number of spikes and average firing frequency. In response to pairs of action potentials elicited at variable frequency (from 10 to 200 Hz), simulation showed a rather homogeneous behavior independently from the excitatory, inhibitory (E/I) balance (black trace Fig. 8C). Of note, in case of null inhibition the first spike delay was anticipated, and the I/O frequency curve was poorly affected by sevoflurane (Fig. 8D). Conversely, similarly to experimental observations, the first spike delay was prolonged by sevoflurane proportionally to the number of active GoCs (Fig. 8C), while I/O frequency curve showed a linear tendency with a slope strongly decreased by sevoflurane. Finally, the number of total emitted spikes in response to pairs of stimuli was significantly reduced in all the combination of E/I balance where there was at least one active GoC (data not shown). Interestingly, by taking into account the number of emitted spikes, sevoflurane reduced the total amount of emitted spikes independently from the number of inhibitory inputs (Fig. 8C).

## Discussion

In this work we have analyzed the impact of sevoflurane, a general anesthetic widely employed in the clinical practice, on neurotransmission properties in a reduced model of brain circuit, the cerebellar cortical microcircuitry. By combining experimental observations with simulations made through a biologically realistic mathematical model, we have dissected the cellular and molecular determinants of the effect of the anesthetic.

Several studies have shown that volatile anesthetics interfere with GABA-mediated synaptic machinery^3,4^ by acting primarily on the postsynaptic side. Sevoflurane increases the total charge transfer through the reduction of peak amplitudes and by slowing current decay^41,42^. Notably, in hippocampal and cortical preparations sevoflurane increases the frequency and, in some degree, the peak amplitude of both sIPSCs and TTX-insensitive miniature IPSCs (mIPSCs), raising the doubt that changes in the presynaptic release machinery could also occur^43^. Our findings show that presynaptic changes are supported by (i) an increase of sIPSCs frequency, (ii) an acceleration of current kinetics, (iii) an increase of peak currents and (iv) a decrease of the eIPSCs paired pulse ratio. Nevertheless, we could also observe a slowing of eIPCSs decay strongly pointing to the concurrent modifies of postsynaptic kinetics. Our hypothesis is supported by mathematical simulations revealing that the effects of sevoflurane observed experimentally could be reproduced only by simultaneously adjusting postsynaptic kinetics and presynaptic release. The discrepancy between our findings and published data could partially reside in the amplification of postsynaptic integration caused by the increased neuronal excitability. Additionally, halogenated anesthetics positively modulate two-pore domain potassium channels^44^, which are expected to lower resting membrane potential^45^, counteracting the increased release probability.

The difference between sIPSC and eIPSC decay changes induced by sevoflurane could originate from variable amounts of neurotransmitter released in response to the activation of a variable number of fibers, which then accumulate in the glomerular space^46^. At the same time, the relative smaller changes induced by sevoflurane on IPSCs decays compared to previously reported results^3,4^ could be due to the small size of the GoC-GrC synapse and to the small amount of neurotransmitter. Halogenated anesthetics can also increase GABAergic neurotransmission through extrasynaptic or tonic mechanisms^47^. The cerebellar GrCs show both phasic and tonic GABAergic currents regulating GrCs repetitive discharge^48^ and decreasing GrCs excitability^49^ respectively. The increase in GrCs excitability suggests that the application of sevoflurane potentiates the phasic and transient component rather than the tonic GABAergic inhibition which, by contrast, should decrease GrCs excitability. The potentiation of GABAergic inhibition together with the depression of excitatory NMDA currents observed in the presence of sevoflurane bring about a reduction of the temporal summation which prevents the generation of GrCs repetitive firing rarely occurring with low frequency input stimuli. Additionally, the fast, transient sodium currents have been shown to be inhibited by halogenated anesthetics^6,50^. However, the specific isoforms affected by halogenated anesthetics^51^ are not expressed in the GrCs^52^. Mathematical modeling revealed that the persistent component of sodium current, showing small amplitudes and markedly impacting membrane excitability due to the high GrC input resistance^53^, can indeed alter GrC firing threshold through an increase of conductance or through changes of voltage sensitivity of activation and inactivation.

The marked increase of GoC inhibition had a major role in dampening membrane potential. However, a decreased temporal summation following the block of NMDA currents can contribute to enhance the effect of the anesthetics in the near threshold regime. We have recently shown how desflurane, a general anesthetic belonging to the same chemical family of sevoflurane, alters the neurotransmission in the cortical cerebellar circuit by estimating the changes in Mutual Information (MI) between mfs and GrCs^33^. Accordingly, sevoflurane alters intrinsic excitability and potentiates GABAergic neurotransmission, whilst inhibits glutamatergic NMDA activity which was unaffected by desflurane^33^. This discrepancy in the modulation of NMDA currents could account for some of the differences evidenced in the clinical practice between the two compounds. For instance, sevoflurane shows a slower recovery phase, that could be attributed to the block of receptors activity persisting after the complete removal of the anesthetic (Fig. 5C). Additionally, since the hypoactivation and a reduced expression of NMDA receptors has been reported to correlate with schizophrenia^54^ and with the emergence of hallucinations^55^, the observed changes in NMDA activity are suitable to explain dreamlike effects such as delirium and agitation that are commonly reported in patients recovering from sevoflurane anesthesia^56^. Volatile anesthetics are in fact a risk factor, for postoperative delirium, an often-underdiagnosed condition, with potentially severe side effects for the patient^57^. Furthermore, the different effect of sevoflurane and desflurane on NMDA receptors could explain the different cognitive impact and potentially lower incidence of delirium between the two compounds. However, it is still debated if it translates into a measurable difference in terms of postoperative cognitive disorders.

We have shown that sevoflurane alters the capability of transferring information between neurons at the cerebellar input stage without silencing firing activity. As in the case of desflurane, the potentiation of GABAergic inhibition and the increased intrinsic excitability, together with the block of NMDA dependent excitatory neurotransmission, lead to a global reduction of action potential generation yielding a more regular firing activity. Interestingly, the linear dependency of the I/O curve shows a lower slope markedly impacting GrCs responses to repetitive high frequency stimulation. These mechanisms, as in the case of desflurane^33^, could produce a significant reduction of the amount of information transferred between neurons.

Sevoflurane markedly reduced and regularized neuronal spiking activity (see Fig. 2). This effect modulates the communication code rather than inducing an unspecified silencing of the neuronal activity. The output becomes less “rich”, an indication of a reduced capability to convey information^33^, in turn related to the reduction of alternative active states. This effect resulted from the concomitant changes of synaptic transmission and post-synaptic excitability. Sevoflurane potentiated GrCs capability to generate action potentials, an essential condition to translate the outcome of synaptic integration in fine tuning of output spikes. Concomitantly, by increasing the inhibitory peak current in response to an increased vesicles release, sevoflurane prevented the post-synaptic membrane depolarization to enter into the repetitive firing regime. The instantaneous increase in synaptic inhibition counteracted the enhanced intrinsic post-synaptic excitability resulting in a reduction of the number of elicited spikes. The input–output frequency relationship was in fact remarkably reduced.

The effect of sevoflurane on neuronal intrinsic excitability could lead, in a predisposed subject, to electrically-induced seizures. One of the primary concerns for providing anesthesia to epileptic patients is the tendency of general anesthetics to favor seizure activity and to interact with antiepileptic drugs^10^. The clinical intervention is normally not required in healthy patients probably due to the balance between the increased synaptic inhibition and the increased neuronal excitability. These effects may also be responsible for delirium and agitation in the recovery from general anesthesia which are often encountered in the pediatric and adolescent subjects^11^. It should also be noted that in young population, GABAergic currents might have depolarizing effects generating hyperexcitatory behaviors^58^. In any case, the increased inhibitory synaptic transmission induced by sevoflurane could contribute to generate the increased brain metabolism observed in anesthetized mice^59^.

Recent experimental evidences have shown that the cerebellum is involved in the integration of cognitive processes^20^. Moreover, thalamic and sub-thalamic circuits, which are known to be deactivated during anesthesia, are tightly bi-directionally connected with the cerebellar circuit^60^. The cerebellum also shows low-frequency mechanisms favoring the communication with thalamic and cortical areas. The cerebellum thus appears a suitable candidate for contributing to sensory and cognitive perception changes induced by anesthesia^2^ and indeed it gains control of the rich-club networks controlling the wakefulness/ unconsciousness switch in deep sleep and propofol-induced general anesthesia^22–24^. Sevoflurane, analogously to desflurane, by reducing the information transfer thorugh the cerebellar circuit may disrupt the communication in the cerebello-thalamo-cortical loop. These data envisage a picture in which the cerebellar activity could be altered during anesthesia as suggested by the reduction of the cerebral blood flow observed during anesthesia with fMRI and PET studies^17^. Moreover, the decrease of the frequency of spontaneous cerebellar activity during anesthesia^18^ along with the appearance of coherent oscillation observed in anesthetized mice and the decrease in the cerebellar entropy^19^, could indicate that the integration of sensory and cognitive processes taking place in the cerebellum are severely modified during general anesthesia. In a broader context, by exerting a millisecond control on output spikes, the cerebellum may help in maintaining the continuity of reality perception which cannot be accounted for only considering the frequency range of cerebrocortical cognitive processing (50–100 ms). Finally, the impact of sevoflurane on cerebellar activity may be involved in the slow return to full mobility during recovery. Although the detrimental effects of anesthesia on cognition are well described^61^, the return to normal movement and the related recovery of cerebellar functions are less studied. The cerebellum is central to most movement-related functions and most importantly to equilibrium and memory. Given the importance of NMDA activity in the induction and expression mechanisms of several forms of plasticity, the observed longer kinetics of NMDA recovery from sevoflurane application could contribute to yield the learning impairment observed in patients during the post-operative recovery from sevoflurane anesthesia. Additionally, sevoflurane has been shown to induce amnesia, hypnosis and immobility, which have indeed been recently correlated with cerebellar lesions^62^, cerebellar structural variations^63^ and with an increased inhibition by Purkinje cells during the perfusion of general anesthetics^64^. The effects provoked by sevoflurane could be therefore potentiated by the altered cerebellar activity. A fine titration of the level of anesthesia, through a better understanding of cellular mechanisms that regulate cerebellar activity and its modulation by sevoflurane and other halogenated compounds, may lead to a reduced incidence of post-operative cognitive dysfunction. Given these results, the involvement of cerebellar circuits during anesthesia could be further investigated with newer perspectives.

The use of mathematical models to reproduce neuronal behavior is one of the most promising tools to explore the pharmacology of neural circuits. We provide evidence that by combining experimental findings and biologically realistic models the activity of full circuits can be reliably reproduced with molecular precision. This approach is an additional tool to proficiently investigate neurotransmission and can generate predictions of neuronal functions by exploring conditions that can be hardly tested with experimental methods. The assembly in fact of modelled neurons and synapses in large-scale networks can provide realistic simulations of physiological and pathological conditions of neuronal cohorts allowing the testing of new molecular compounds and the exploration of new therapeutic strategies.

In conclusion, these results identify important changes in cerebellar granule cell synaptic activation and excitation in the presence of the general anesthetic, sevoflurane, implying that changes will reverberate on local computation in the granular layer^15^. In cascade, this will alter adaptive filtering at the input stage and reverberate onto the entire cerebellar network. These results prompt for further consideration of the role of cerebellum in regulating the wakefulness/ unconsciousness switch and in mediating the action of general anesthetics in large-scale brain networks.

## Methods

Experiments were performed using Sprague–Dawley rats at postnatal day P17–P24 [internal breeding, Charles-Rivers (Calco, Lecco, Italy)]. All experiments were conducted in accordance with international guidelines from the European Community Council Directive 86/609/EEC on the ethical use of animals and were approved by the Ethical committee of the Italian Ministry of Health and by the Ethical Committee of the University of Modena and Reggio Emilia. Furthermore, the study was carried out in compliance with the ARRIVE guidelines (http://www.nc3rs.org.uk/page.asp?id=1357).

Animals (n = 28) were chosen independently from gender and a total number of 50 cells were employed to perform this research.

### Cerebellar slices

Parasagittal cerebellar slices were obtained as described previously^65^. Briefly, rats were deeply anesthetized with isoflurane (Sigma-Aldrich, Saint Louis, MO, USA) and decapitated. The cerebellum was removed, the vermis isolated and fixed on a vibroslicer stage (VT1000S, Leica Microsystems, Nussloch, Germany) with cyanoacrylic glue. Acute 200-µm thick slices were cut in cold cutting solution containing (in mM): 130 K-gluconate, 15 KCl, 0.2 EGTA, 20 HEPES and 10 glucose, pH adjusted at 7.4 with NaOH. Slices were incubated at 32 °C for at least 1 h before recordings in oxygenated extracellular Krebs solution containing (in mM): 120 NaCl, 2 KCl, 1.2 MgSO_4_, 26 NaHCO_3_, 1.2 KH_2_PO_4_, 2 CaCl_2_, 11 glucose (pH 7.4 when equilibrated with 95% O2 and 5% CO_2_). Slices were then transferred to a recording chamber on the stage of an upright microscope (Zeiss Axioexaminer A1, Oberkochen, Germany) and perfused at 1.5 ml min − 1 with oxygenated Krebs solution maintained at 32 °C with a thermostatic controller (Multichannel system, Gmbh, Reuntlingen, Germany). Slices were immobilized with a nylon mesh attached to a platinum Ω-wire.

### Patch-clamp recordings

Whole-cell recordings from GrCs were obtained with the patch-clamp technique^66^ by using an Axopatch 200B amplifier (Molecular Devices, Union City, CA, USA) (− 3 dB; cut-off frequency = 2 kHz). Recordings were digitized at 20 kHz using pClamp 9 (Molecular Devices) and a Digidata 1322A A/D converter (Molecular Devices). Patch pipettes were made with a vertical puller (model PP-830, Narishige, Tokyo, Japan) from borosilicate glass capillaries and filled with the following solution (in mM): 126 K-gluconate, 8 NaCl, 15 glucose, 5 HEPES, 1 MgSO_4_, 0.1 BAPTA-free, 0.05 BAPTA-Ca^2+^, 3 ATP, 100 µM GTP; pH adjusted to 7.2 with KOH. This solution maintained resting free-[Ca^2+^] at 100 nM and pipettes had a resistance of 7–10 MΩ before seal formation.

Mossy fibers (excitatory inputs to GrCs; Fig. 1A) were stimulated with a bipolar tungsten electrode (Clark Instruments, Pangbourne, UK) via a stimulus isolation unit. Stimulation intensity (± 5–15 V; 100 μs) was raised until the excitatory synaptic activity generated at least 1 spike in GrCs at a membrane potential between − 55 and − 65 mV (mean − 59.2 ± 1.9 n = 14). From a comparison with previous data and mathematical models^65^, in these conditions from 2 to 4 mossy fibers were stimulated per GrC depending on the level of synaptic inhibition. Excitatory Post-Synaptic Potentials (EPSPs) were analyzed in terms of rise time, amplitude and total depolarization calculated as the integral of the membrane depolarization between the onset and 50 ms from the synaptic stimulation. The total depolarization was used as an index of membrane depolarization changes in different conditions.

Golgi cell axon bundles (inhibitor inputs to GrCs; Fig. SM-1A) were stimulated via bipolar tungsten electrode with two stimuli at 50 Hz repeated at 0.1 Hz. Paired inhibitory post-synaptic currents (IPSCs) were detected in voltage-clamp configuration by holding neurons at 0 mV and appeared as positive deflections given that the chloride reversal potential was set at about − 60 mV. Evoked IPSCs (eIPSCs) were isolated by adding to the bath solution 10 µM NBQX (Tocris Bioscience, Bristol, UK) and 25 µM D-APV (Tocris Bioscience, Bristol, UK) to block glutamate AMPA and NMDA receptors, respectively. The NMDA current was isolated by voltage clamping GrCs at − 40 mV and in the presence of 10 µM SR9519 (Gabazine; Tocris Bioscience, Bristol UK), a selective GABA-A receptor inhibitor. Peak amplitude, time to peak, rise time from 10 to 90% of peak amplitude (rise_10–90_) were computed. The decay components of synaptic currents were approximated by mono-exponential fitting between the peak and the baseline and time constant (τ) was evaluated. Total charge transfer was calculated by measuring IPSCs area to estimate potential differences in the synaptic release probability. At the end of some experiments IPSCs were blocked with 10 µM Gabazine.

In patch-clamp recordings, membrane currents can be influenced by modifications of series resistance, mainly due to pipette tip clogging (access resistance). To ensure that series resistance remained stable throughout the experiments, we analysed current relaxation induced by a 10 mV step from the holding potential (0 mV and − 70 mV for IPSCs and EPSCs, respectively). According to previous reports, the transients were reliably fitted with a mono-exponential function yielding membrane capacitance of 2.8 ± 0.3 pF, input resistance of 1.9 ± 0.1 GΩ, and series resistance of 17.7 ± 0.4 MΩ (n = 28). These parameters were monitored during the perfusion of anesthetic and none of them was significantly changed by sevoflurane. Furthermore, the “resting” membrane potential was monitored throughout the current clamp recordings. The intrinsic excitability was evaluated by measuring the amount of current injected to elicit action potential from a resting membrane potential of − 60 mV, while the spike after-hyperpolarization was measured as the difference between the spike threshold and the minimum level of membrane potential after the spike.

### Perfusion with anesthetic

Aqueous anesthetic solution was prepared to obtain a final concentration in the recording chamber compatible with previously reported data. Sevoflurane concentration has been used typically in the range of 0.2–1.5 mM (e.g.^41,51^). This range could be accounted for by differences in the tissue preparation, perfusion system and actual anesthetic concentration in the tissue which is affected by the highly hydrophobic nature of the molecule.

The desired concentration was obtained by adding 2 ml of sevoflurane (Baxter, Deerfield, IL, USA) directly to the extracellular solution up to a total volume of 50 ml in a closed vial (4% vol/vol). Vials were shaken and left 60 min to equilibrate before filtering (0.5 μm diameter) and adding the supernatant directly to the gravity-driven perfusion system. In some experiments (n = 4) the anesthetic concentration was determined by means of gas chromatography coupled with mass spectrometry (GC–MS). A GC 7890A (Agilent Technologies, Waldbronn, Germany), coupled with a single quadrupole 5975C TAD Series GC/MSD system (Agilent Technologies). Identification of sevoflurane was achieved by using mass fragmentation data and comparison with the literature. According to GC–MS quantification, the concentration of sevoflurane in the reservoir was 0.017 ± 0.003% vol/vol (n = 4; *p* < 0.01) corresponding to 1.3 ± 0.2% mM (n = 4; *p* < 0.01), while the solution directly taken from the recording chamber contained sevoflurane at a concentration of 0.014 ± 0.0003% vol/vol (n = 4; *p* < 0.01) corresponding to 1.1 ± 0.02 mM (n = 4; *p* < 0.01).

### Mathematical modeling

#### Single cell and synaptic models

The synaptic models of single neurons were adapted from the original scheme reported in^65^ and could reproduce the kinetics and size of the postsynaptic currents during repetitive synaptic transmission at the different synapses. These models accounted for vesicular dynamics, neurotransmitter spillover and receptor gating (including multiple closed, desensitized and open states) but not for quantal release mechanisms. The dynamics of synaptic responses were fully determined by the kinetic constants of synaptic and neuronal models. Axonal conduction times were considered negligible and transmission delay was set 1 ms for all the synapses.

In order to conform to in vivo conditions, all models were adapted from their original temperature T_orig_ to T_sim_ = 37 °C using the correction factor Q_10_ = (T_sim_ – T_orig_)/10. We have used: Q_10_ = 3 for ionic channel gating, Q_10_ = 2.4 for receptor gating, Q_10_ = 1.5 for ionic channel permeation, Q_10_ = 1.3 for neurotransmitter diffusion, Q_10_ = 3 for Ca^2+^ pumps and buffers, Q_10_ = 1.3 (GrC) or 1.7 (GrC) for intracellular Ca^2+^ diffusion. Following adaptation at 37 °C, the models were in matching with recordings at this same temperature (data not shown).

The GrC model was adapted from^65^ by applying appropriate Q_10_ corrections. In addition, the GABA leakage conductance was increased by two times (60 µS/cm2), the inward rectifier K^+^ conductance was increase by 1.5 times (1350 µS/cm2) and the leakage reversal potential was adjusted to restoring resting potential to − 70 mV. With this asset, the GrC model properly reproduced responses to current injection at 37 °C (data not shown) and spike trains observed in vivo.

The GoC model was adapted from^67^ by applying appropriate Q_10_ corrections. Without needing any further change, the GoC model properly reproduced responses to peripheral stimulation observed in vivo.

All the results shown in the manuscript have been obtained by setting the temperature in the simulation at 30°, which accounts for a thermal dispersion of the solution in the recording chamber (from 32° at the border where the perfusing syringe is located to about 30° in the middle of the chamber).

The glutamatergic mf-GrC synapses take part to the formation of the cerebellar glomerulus and activate AMPA and NMDA receptors. The release, diffusion and ionic receptor mechanisms were the same reported by^65^. Using a probability of release of 0.6, the model was able to faithfully reproduce postsynaptic currents recorded at 37 °C in vitro^65^ and in vivo^68^. The time constant of the recovery from depression, τ_REC_ = 8 ms, was derived from in vivo measurements and allowed to reproduce natural dynamics of short-term plasticity (the time constants of presynaptic facilitation and vesicle inactivation were set to τ_facil_ = 5 ms and τ_I_ = 1 ms, respectively).

The mf-GoC synapses are similar in several aspects compared to the mf-GrC synapses. They are also located within the cerebellar glomerulus and are glutamatergic activating both AMPA and NMDA receptors. The mf-GoC synapse was adapted from the mf-GrC synapse model (see above) to reproduce a peak of postsynaptic current of − 66 pA. Release probability and vesicle cycling parameters were set at the same values as at the mf-GrC synapse.

The GrC-GoC synapses are formed by PFs onto GoC apical dendrites in the molecular layer. These glutamatergic synapses activate AMPA, NMDA and kainate receptors. During repetitive stimulation, the AMPA current shows synaptic depression while the kainate and NMDA currents show slow temporal summation. AMPA and NMDA currents were taken from the MF-GrC synapses and the kainate receptor current was modified from the AMPA kinetic scheme. Release probability was 0.1 and vesicle cycling parameters were set at the same values as at the MF-GrC synapse. The AA contacts GoC basolateral dendrites in the granular layer; these synapses activate AMPA and NMDA only; their maximal conductance was estimated to be ~ 2 times higher than AMPA and NMDA currents of PF-GoC synapses. Also, in this case AMPA and NMDA currents were taken from the MF-GrC synapse; release probability and vesicle cycling were set at the same values, too.

The GoC-GrC synapses are GABAergic and impinge on GrC dendrites within the glomerulus. The GABA-A receptor schemes comprised channels with fast (α1) and slow (α6) kinetics and GABA spillover generating the transient and sustained components of inhibition observed experimentally. In order to account for experimental results, the parameters describing presynaptic dynamics were: release probability = 0.35, τ_REC_ = 36 ms, τ_facil_ = 58.5 ms and τ_I_ = 0.1 ms, respectively.

### Statistical analysis

Data are reported as means ± standard error of the mean (SEM). All the statistical comparisons were done using Student’s t-test.

## Supplementary information

Supplementary information.
