# Supplementary material for: The effects of the general anesthetic sevoflurane on neurotransmission: an experimental and computational study
Source: Sci Rep. 2021 Feb 22;11:4335. doi: 10.1038/s41598-021-83714-y (PMC7900247; doi:10.1038/s41598-021-83714-y)
Supplement: Supplementary file 1 — Supplementary information. [file 41598_2021_83714_MOESM1_ESM.pdf]

SUPPLEMENTARY MATERIAL TO THE MANUSCRIPT:

"The effect of the general anesthetic sevoflurane on neurotransmission: an experimental and computational study"

Jonathan Mapelli1,2*†, Daniela Gandolfi1†, Enrico Giuliani3†, Stefano Casali4, Luigi Congi1, Alberto Barbieri3, Egidio D'Angelo4,5 and Albertino Bigiani 1,2

1 Department of Biomedical, Metabolic and Neural Sciences, University of Modena and Reggio Emilia, Modena, I-41125, Italy; jonathan.mapelli@unimore.it, daniela.gandolfi@unimore.it, albertino.bigiani@unimore.it
2 Center for Neuroscience and Neurotechnology, University of Modena and Reggio Emilia, Modena, I-41125, Italy; jonathan.mapelli@unimore.it, albertino.bigiani@unimore.it
3 Department of Medical and Surgical Sciences for Children and Adults, University of Modena and Reggio Emilia, Modena, I-41125, Italy,
4 Department of Brain and Behavioral Sciences, University of Pavia, I-27100, Pavia, Italy
5 Brain Connectivity Center, IRCCS Mondino Foundation, I-27100, Pavia, Italy

Figure SM-1

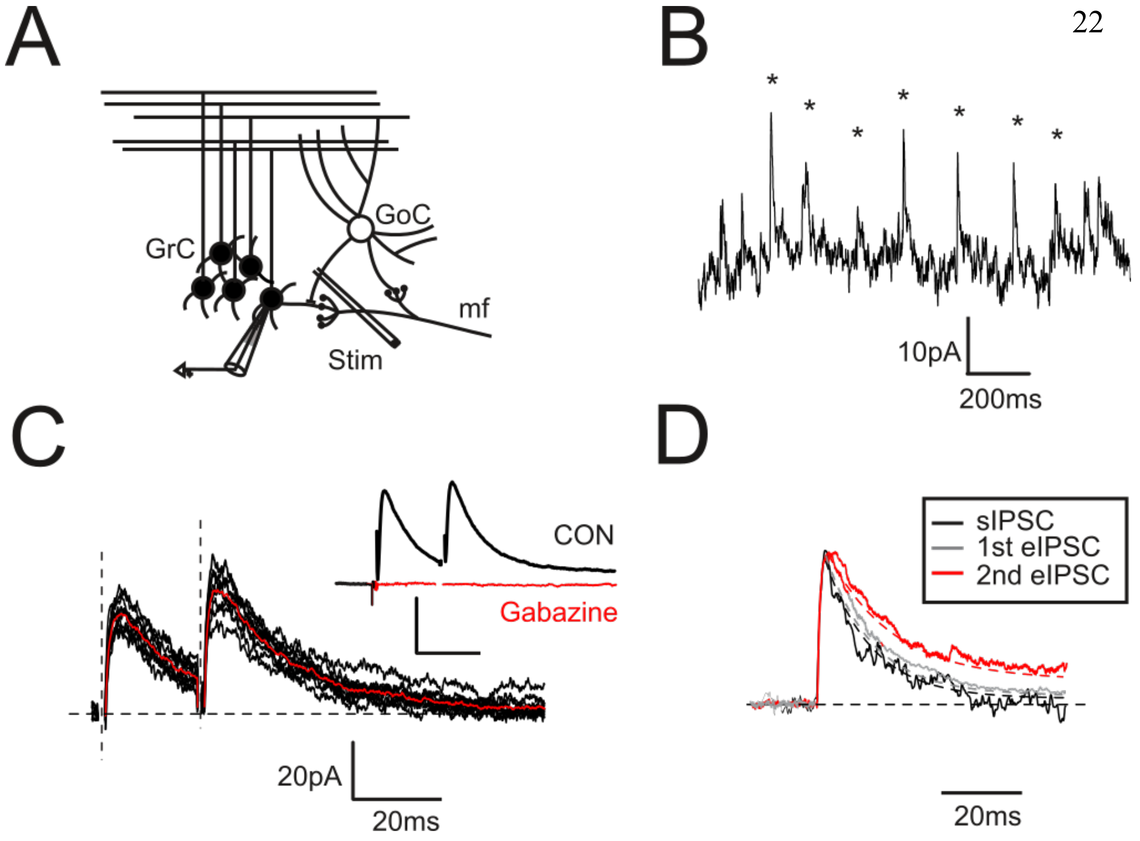

Figure SM-1. Inhibitory neurotransmission and sevoflurane. A. The stimulating electrode (stim) is placed in the surrounding of the recorded GrC in order to elicit action potentials in the axonal plexus of the GoC. B. The spontaneous activity (spontaneous Inhibitory Post-Synaptic Currents, sIPSCs) recorded from a GrC voltage clamped at 0 mV reflects the autorhythmic discharge of GoCs. The asterisks indicate spontaneous events evenly spaced and induced by the regular firing of a GoC. C. eIPSCs evoked by 2 pulses at 50 Hz (vertical lines). eIPSCs were elicited in the supra-minimal range so that sIPSCs amplitudes (9.4 ± 1.3 pA; n = 9 cells and n = 198 events) were significantly smaller than eIPSCs (28.3 ± 5.9 pA, $p < 0.01$; n = 9). The red trace shows the average of 10 consecutive acquisitions (black traces). Inset. The application of Gabazine (red) completely abolished eIPSCs (n=5). D. Normalized spontaneous (black trace) and evoked IPSCs (gray trace 1st response, red trace 2nd response). Note the similar kinetics of the 1st evoked and spontaneous IPSC. The 2nd eIPSC has a longer decay phase probably due to the neurotransmitter accumulation in the glomerular space.

Supplementary tables

	Control	Sevoflurane	Wash out
V_0	-63.2 ± 2.1 mV	-61.9 ± 2.2 mV	-62.7 ± 1.9 mV
R_{input}	1.9 ± 0.1 GΩ	1.9 ± 0.4 GΩ	1.8 ± 0.3 GΩ

Table SM-1. Zero-current potential (V0) and Input Resistance (Rinput) of granule cells in control condition and during perfusion with sevoflurane (n = 7).

Parameter	**CONTROL**	**SEVOFLURANE**
Release probability	***0.35***	***0.5***
α1 GABA conductance (pS)	*918.807*	*2500*

α1 GABA conductance (pS)	*132.842*	*800*
a1 activation kinetics	*0.414*	*0.514*
b1 activation kinetics	*0.03*	*0.3*
R1 activation kinetics	$7 * 10^{-4}$;	$7 * 10^{-3}$;
R2 activation kinetics	*0.14208*	*1.4208*
d2 activation kinetics	*3,4898*	*10*
d3 activation kinetics	*21,703*	*30*
Koff deactivation kinetics	*2*	*1*

Table SM-2 Synaptic model parameters
